# Supplementary figures and images for: Functional architecture underlying binocular coordination of eye position and velocity in the larval zebrafish hindbrain
Source: BMC Biol. 2019 Dec 29;17:110. doi: 10.1186/s12915-019-0720-y (PMC6936144; doi:10.1186/s12915-019-0720-y)

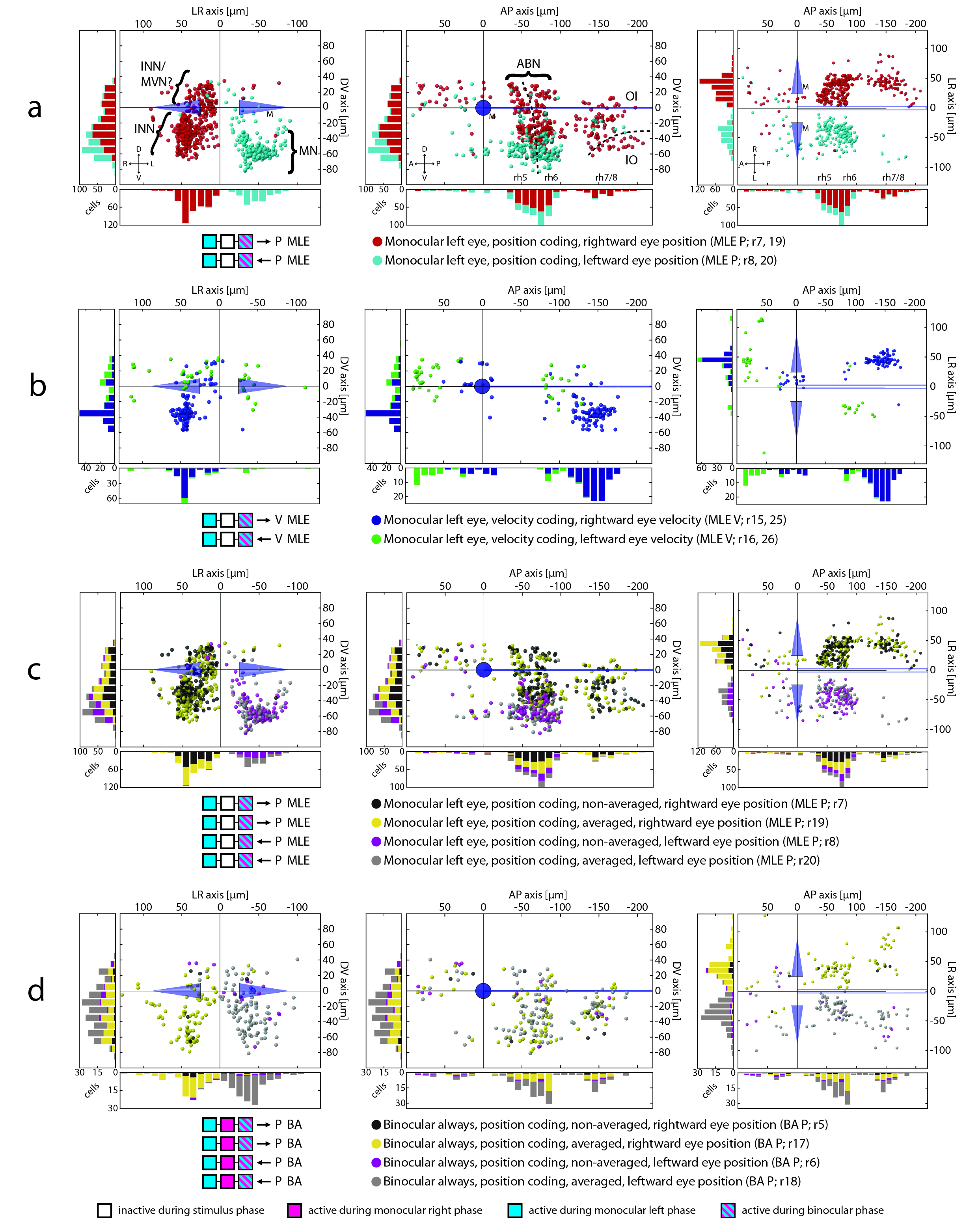

Supplement: Supplementary file 2 — Additional file 1: Figure S1. Monocular left eye neurons and examples for single regressors. Additional regressor cell maps for Fig. 3. a-d: Transversal, sagittal and dorsal views for MLE and BA neurons in the hindbrain. The MLE maps in a-b correspond to neurons which are mirror-symmetric with respect to the neurons plotted in Fig. 3a-b. The plot in c show the lack of clustering of neurons identified with two similar, but slightly different, regressors (averaged and non-averaged regressors). Plot d shows the difference for BA P neurons due to the different motor range explored during the monocular and binocular stimulation phases (Methods). A: anterior; ABN: nucleus abducens; BA: binocular always; D: dorsal; L: left; INN: internuclear neurons; IO: inferior olive; M: Mauthner cells; MLE: monocular left eye; MN: motoneurons; MVN: medial vestibular nucleus; OI: oculomotor integrator; P: position/posterior; R: right; r: regressor; rh 5-8: rhombomeres 5-8; V: ventral/velocity; lines show the rough borders between individual neuronal clusters. [file 12915_2019_720_MOESM1_ESM.tif]

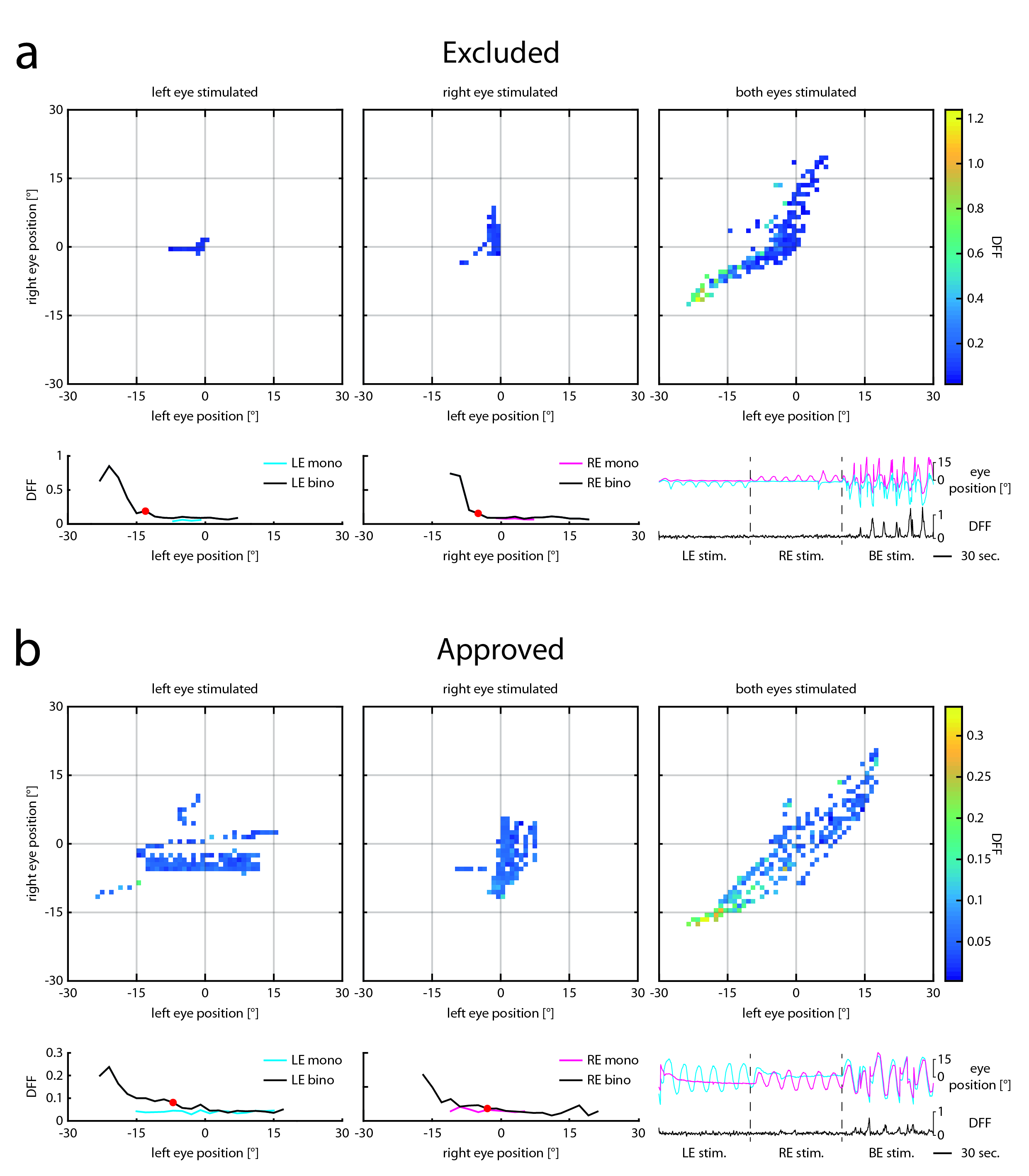

Supplement: Supplementary file 3 — Additional file 2: Figure S2. Firing threshold analysis. Tuning curves during the monocular and binocular stimulus phases for one neuron excluded from further analysis (a) and one neuron included in further analysis (b). a: The upper row shows the neural activity (DFF) color coded during the monocular left eye (left plot), right eye (middle plot) and binocular (right plot) stimulus phases for individual eye position bins. Monocular tuning curves (cyan left eye, magenta right eye) were plotted for the respective monocular stimulus phase and the binocular stimulus phase (black). Only bins with at least three individual data points were used. Red dot shows firing threshold. For this neuron, the eye position never explored the eye position threshold during the monocular stimulus phases, it was thus excluded from further analysis. In the lower right the corresponding eye positions and neural activity (DFF) are plotted versus time. b: Tuning curves and eye positions for one threshold approved neuron. Note that for this neuron, the monocular tuning curves covered the eye position threshold (red dot). [file 12915_2019_720_MOESM2_ESM.tif]

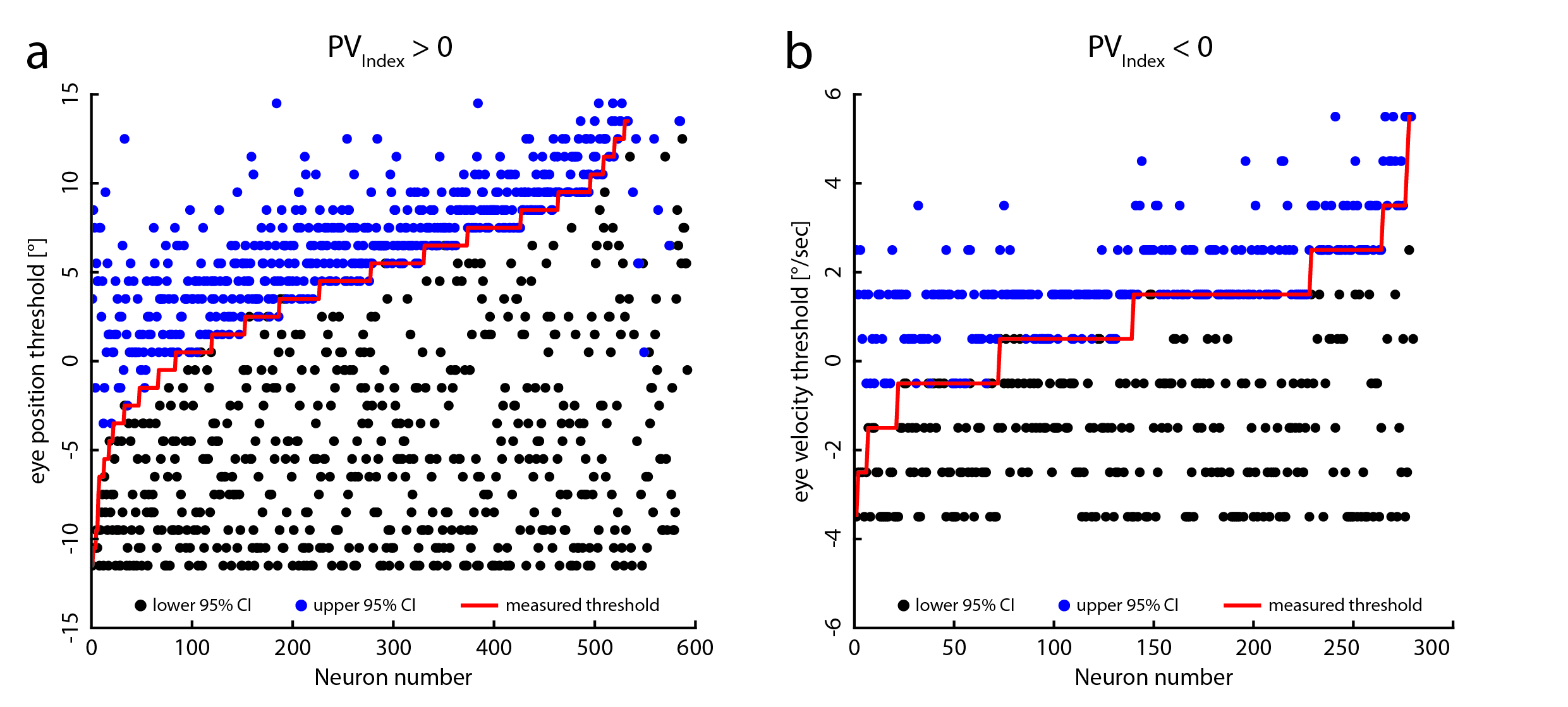

Supplement: Supplementary file 4 — Additional file 3: Figure S3. Firing threshold bootstrap analysis. Bootstrap analysis with 95 % confidence intervals for the threshold estimation. a: Neurons with positive (position coding) PVIndex (n = 592; 533 with identified threshold). b: Neurons with negative (velocity coding) PVIndex (n = 280; 279 with identified velocity threshold). Firing thresholds were pooled in ON direction. Red line shows the measured threshold for each neuron, the blue and black dots the respective upper and lower 95 % confidence bounds for 1000 repetitions. [file 12915_2019_720_MOESM3_ESM.tif]

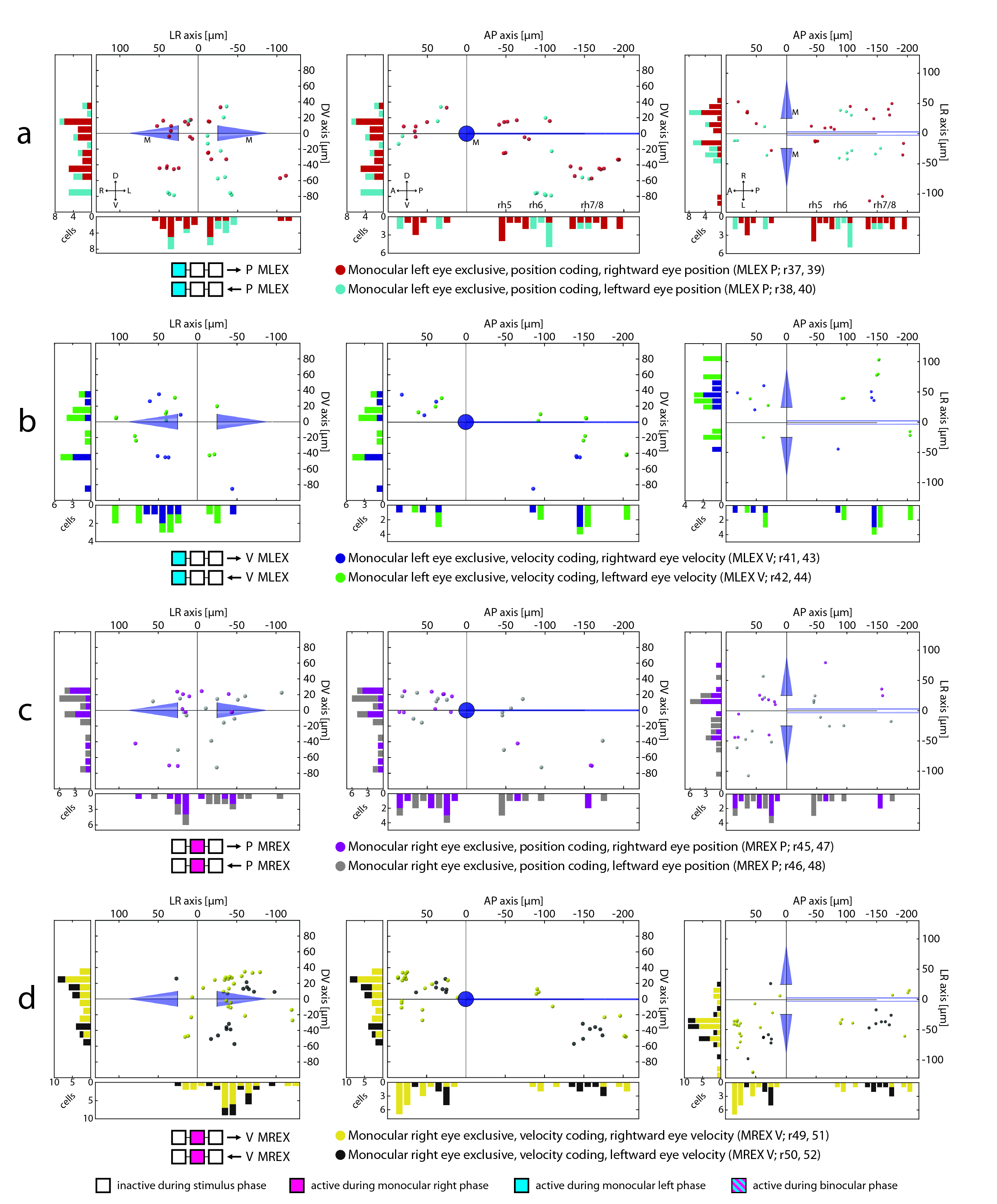

Supplement: Supplementary file 5 — Additional file 4: Figure S4. Cell maps for monocular exclusive neurons. a-d: Transversal, sagittal and dorsal views for MLEX and MREX neurons. A: anterior; D: dorsal; L: left; M: Mauthner cells; MLEX: monocular left eye exclusive; MREX: monocular right eye exclusive; P: position/posterior; R: right; r: regressor; rh 5-8: rhombomeres 5-8; V: ventral/velocity. [file 12915_2019_720_MOESM4_ESM.tif]

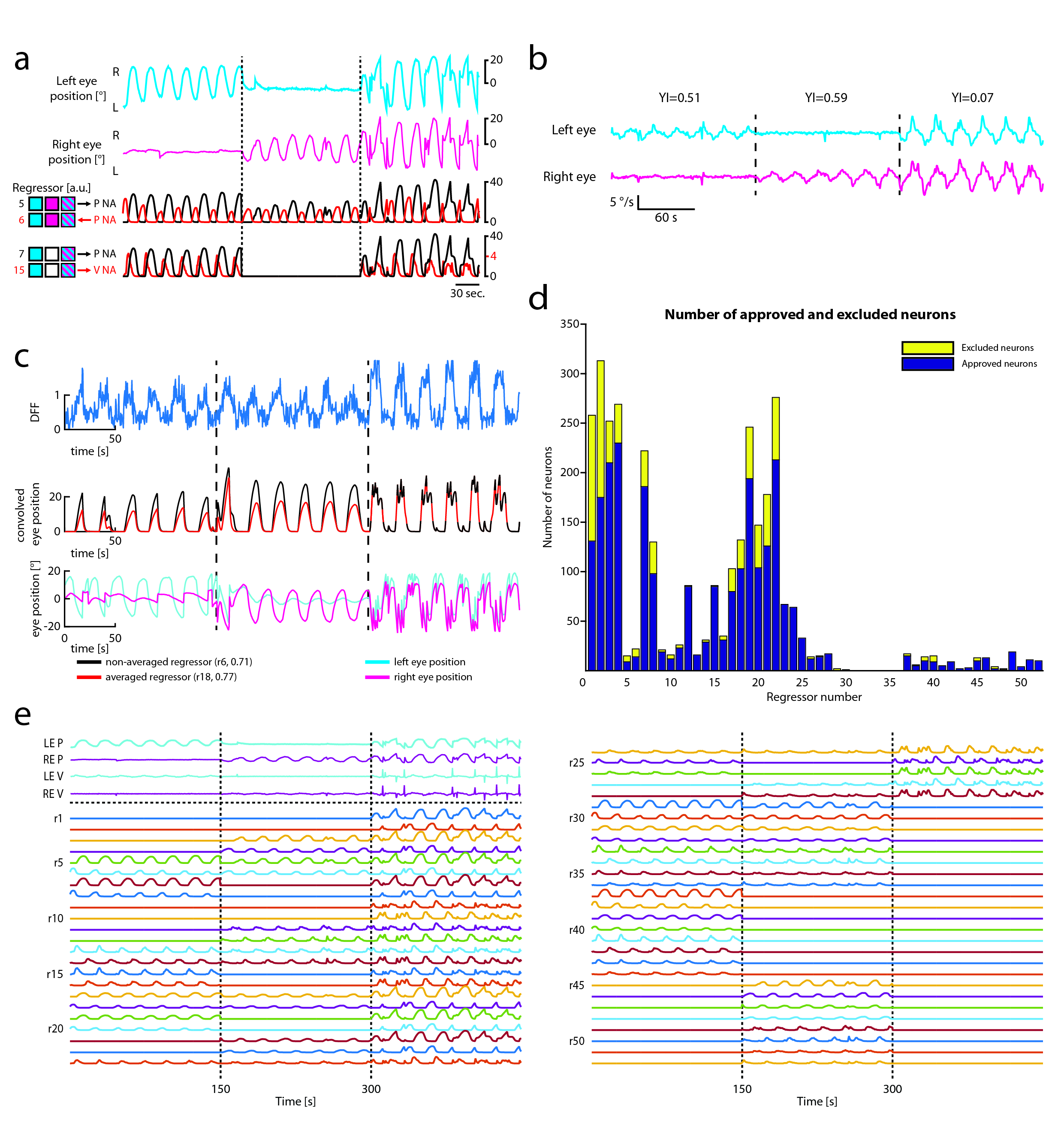

Supplement: Supplementary file 6 — Additional file 5: Figure S5. Methods for monocular/binocular analysis. Additional regressor examples for the monocular/binocular coordination experiment. a: Example regressors and respective eye traces. L: left; NA: non-averaged (see Methods); P: position; R: right; V: velocity; eye traces same as in Fig. 2a’; b: Example eye traces for yoking index exclusion. YI: yoking index; c: Example binocular always (BA) neuron and the highest scoring regressor r6 (non-averaged) with the corresponding averaged regressor (r18) and eye traces they are based upon. d: Overview of all approved and excluded neurons for each regressor based on the firing threshold analysis. e: All derived regressors from recording shown in Fig. 2a-a’. LE: left eye; P: position; RE: right eye; V: velocity; r: regressor. [file 12915_2019_720_MOESM5_ESM.tif]

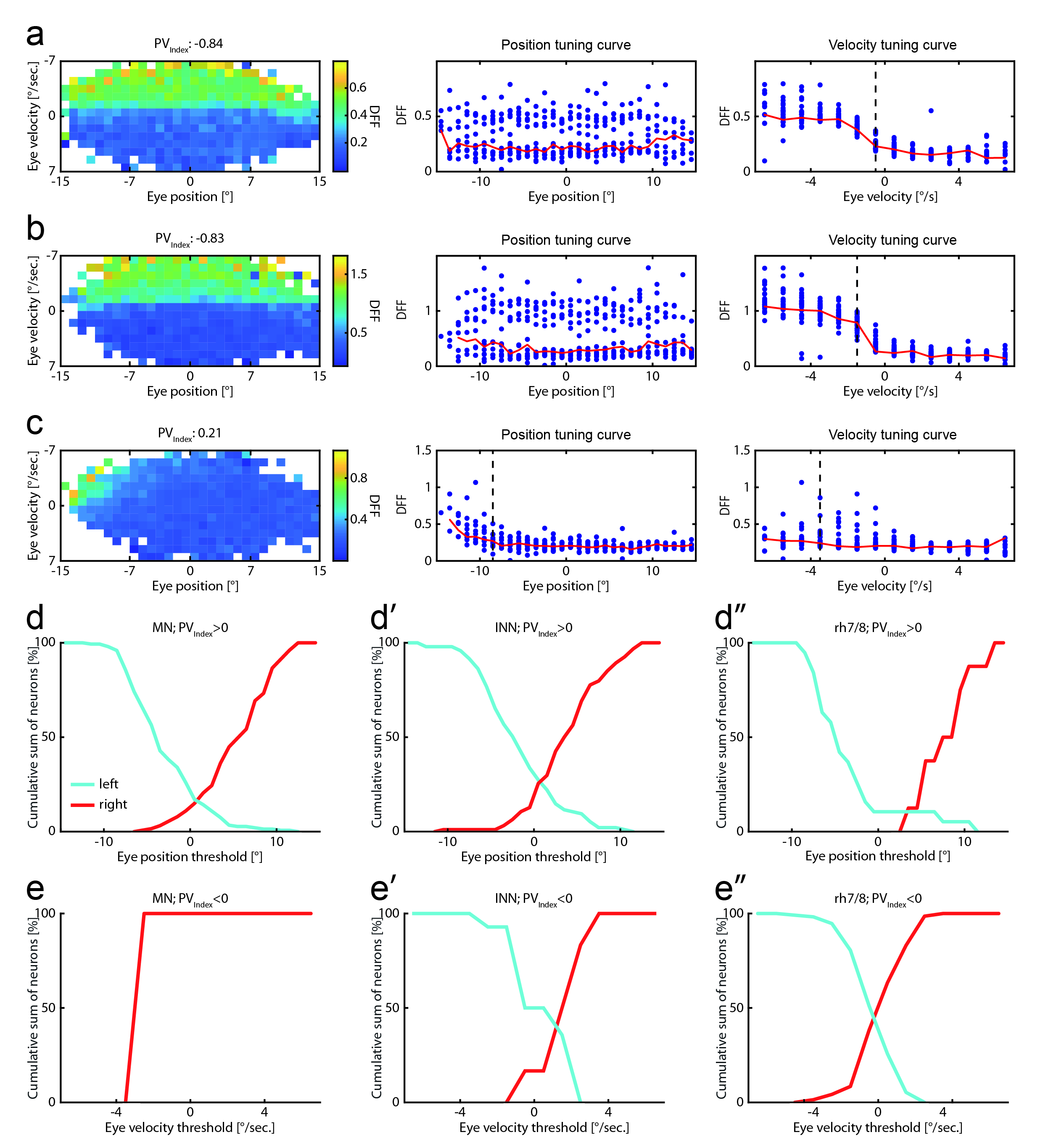

Supplement: Supplementary file 7 — Additional file 6: Figure S6. Additional tuning curves and firing thresholds for different neuron populations. a-c: Additional tuning curve plot same as in Fig. 5. d-d”: Cumulative position threshold plots for position coding neurons (PVIndex > 0) pooled in ON for motoneurons (d, left: 147, right: 127), internuclear neurons (d’, left: 95, right: 94, both based on their anatomical location) and the caudal hindbrain (d”, left: 19, right: 8). e-e”: Cumulative velocity threshold plots for velocity coding neurons (PVIndex < 0) pooled in ON for motoneurons (e, left: 0, right: 1), internuclear neurons (e’: left: 14, right: 6) and the caudal hindbrain (e”: left: 113, right: 71). [file 12915_2019_720_MOESM6_ESM.tif]

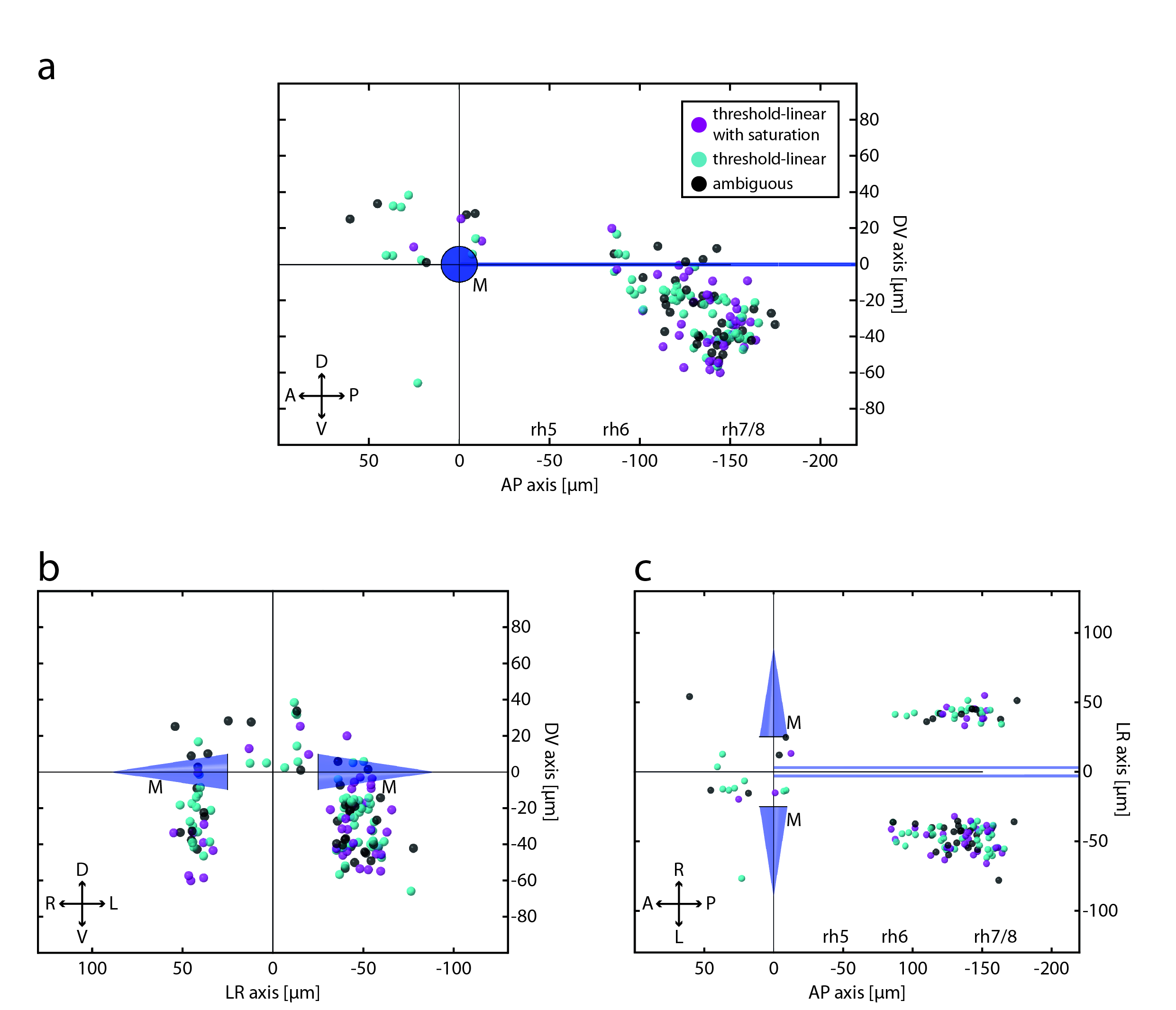

Supplement: Supplementary file 8 — Additional file 7: Figure S7. Velocity neurons with different response profiles show no spatial clustering. a-c: Sagittal, transversal and dorsal view of threshold-linear (n = 60), threshold-linear with saturation (n = 40) and ambiguous (n = 39) neurons (PVIndex < -0.5) color-coded according to their response type. A: anterior; D: dorsal; L: left; M: Mauthner cells P: posterior; R: right; rh5-8: rhombomeres 5-8; V: ventral. [file 12915_2019_720_MOESM7_ESM.tif]

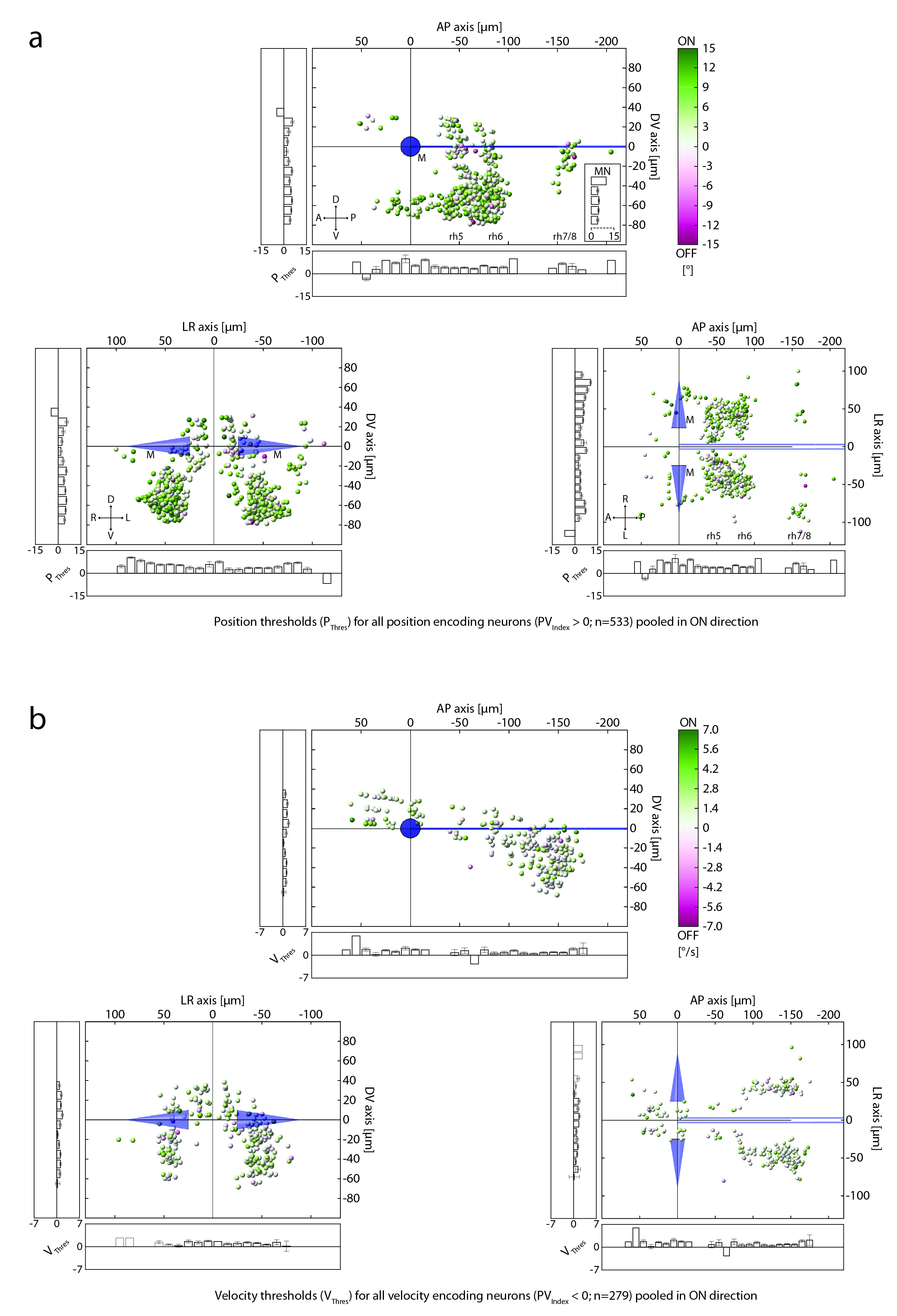

Supplement: Supplementary file 9 — Additional file 8: Figure S8. Position and velocity thresholds. Transversal, sagittal and dorsal views of position and velocity coding neurons color-coded for their thresholds. a: Position thresholds (PThres) color-coded for all position coding neurons (PVIndex > 0) with an identified firing threshold pooled in ON direction (n = 533). Inset shows thresholds for motoneurons based on their anatomical location (no statistical significance was observed: Kruskal-Wallis p = 0.22; n = 2, 41, 98, 89, 43) b: Velocity threshold (VThres) color-coded for all velocity coding neurons (PVIndex < 0) with an identified firing threshold pooled in ON direction (n = 279). [file 12915_2019_720_MOESM8_ESM.tif]
